# Investigating skyrmion stability and core polarity reversal in NdMn_2_Ge_2_

**DOI:** 10.1038/s41598-024-82114-2

**Published:** 2025-01-02

**Authors:** Samuel K. Treves, Victor Ukleev, Andreas Apseros, Jamie Robert Massey, Kai Wagner, Paul Lehmann, Aki Kitaori, Naoya Kanazawa, Jeffrey A. Brock, Simone Finizio, Joakim Reuteler, Yoshinori Tokura, Patrick Maletinsky, Valerio Scagnoli

**Affiliations:** 1 Department of Physics, University of Basel, 4056 Basel, Switzerland; 2 Laboratory for Mesoscopic Systems, Department of Materials, ETH Zurich, 8093 Zurich, Switzerland; 3 PSI Center for Neutron and Muon Sciences, 5232 Villigen PSI, Switzerland; 4 Helmholtz-Zentrum Berlin für Materialien und Energie, D-14109 Berlin, Germany; 5 Department of Applied Physics, University of Tokyo, Tokyo, 113-8656 Japan; 6 Institute of Engineering Innovation, The University of Tokyo, Tokyo, 113-0032 Japan; 7 Institute of Industrial Science, The University of Tokyo, 4-6-1 Komaba Meguro-ku, Tokyo, 153-8505 Japan; 8 PSI Center for Photon Science, 5232, Villigen PSI, Switzerland; 9 ScopeM, ETH Zurich, 8093 Zurich, Switzerland; 10 RIKEN Center for Emergent Matter Science (CEMS), Wako, 351-0198 Japan; 11 Tokyo College, University of Tokyo, Tokyo, 113-8656 Japan

**Keywords:** Materials science, Physics

## Abstract

We present a study on nanoscale skyrmionic spin textures in $\hbox {NdMn}_{{2}}\hbox {Ge}_{{2}}$, a rare-earth complex noncollinear ferromagnet. We confirm, using X-ray microscopy, that $\hbox {NdMn}_{{2}}\hbox {Ge}_{{2}}$ can host lattices of metastable skyrmion bubbles at room temperature in the absence of a magnetic field, after applying a suitable field cooling protocol. The skyrmion bubbles are robust against temperature changes from room temperature to 330 K. Furthermore, the skyrmion bubbles can be distorted, deformed, and recovered by varying strength and orientation of the applied magnetic field. We have used nitrogen-vacancy nanoscale magnetic imaging to estimate and map the magnetic stray fields originating from our $\hbox {NdMn}_{{2}}\hbox {Ge}_{{2}}$ lamella samples and find stray field magnitudes on the order of a few mT near the sample surface. Micromagnetic simulations show an overall agreement with the observed behaviour of the sample under different magnetic field protocols. We also find that the presence of the Dzyaloshinskii-Moriya interaction is not required to reproduce our experimental results. Its inclusion in the simulation leads to a reversal of the skyrmionic object core polarity, which is not experimentally observed. Our results further corroborate the stability and robustness of the skyrmion bubbles formed in ${\hbox {NdMn}_2\hbox {Ge}_2}$ and their potential for future spintronic applications.

## Introduction

Future spintronic device architectures could implement topologically stable spin textures, such as skyrmions^1,2^ to improve the speed and efficiency of magnetic memory devices^3–6^. These topologically non-trivial magnetic textures have been typically observed^7–12^ in systems exhibiting the Dzyaloshinskii-Moriya (DMI) spin-spin interaction^13,14^ — an antisymmetric exchange interaction between neighbouring magnetic moments, which favours non-collinear magnetic structures. The DMI arises from the spin-orbit coupling between the electron spins and the crystal lattice and is allowed for systems with broken spatial inversion symmetry. The DMI can induce a non-collinear spin texture, which in turn can develop into a skyrmion configuration. Bulk DMI can be found in non-centrosymmetric bulk crystals (e.g., MnSi^7^, $\hbox {Cu}_{{2}}\hbox {OSeO}_{{3}}$^15^). Another type of DMI is present in thin film systems^16^, where the interaction is interfacial^17^. Single crystal B20 structures can host skyrmions that are typically only stable in a small range of magnetic fields and below room temperature^7,8,18,19^.

Skyrmion lattices have also been observed above and at room temperature in the absence of a magnetic field in single crystals such as Co-Zn-Mn^10,20^ and Fe-Ni-Pd-P^21^ alloys.

For skyrmion hosting systems to be used for technological applications, they should satisfy a few key criteria^22^. Firstly, they should host stable skyrmions at room temperature, ideally without the need for an external magnetic field to be applied. The skyrmions must also exhibit stability over such timescales to prevent data loss.

The search for candidate materials suitable for such applications has revealed that the presence of topologically non-trivial magnetic configuration also occurs in materials with no DMI. Recent studies have led to the discovery of topological magnetic textures in centrosymmetric non-collinear magnets such as MnNiGa^23^, Fe/Gd^24^, or $\hbox {Fe}_{{3}}\hbox {Sn}_{{2}}$^25^. So-called skyrmion bubbles (SkBs) can be stabilised in these systems, provided that a sizable uniaxial magnetic anisotropy exists in the system that competes with the magnetic dipole interaction^26^. SkBs are topologically equivalent to skyrmions, however, contrary to skyrmions, SkBs can occur in systems that lack DMI. In such systems, only Bloch-type SkBs can form since their Néel-type counterpart requires DMI to be stabilised^27^.

**Fig. 1:**
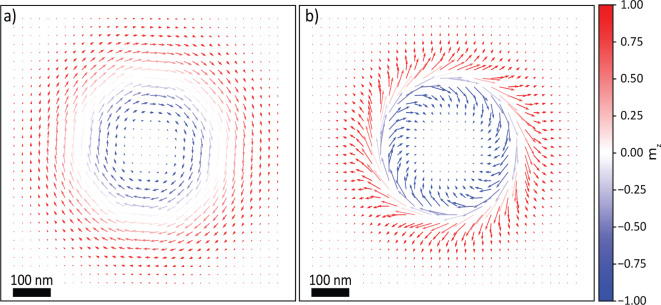
Comparison of the magnetisation configuration for (**a**), a Bloch skyrmion and (**b**), a Skyrmion bubble as obtained from micromagnetic simulations. The colourmap refers to the magnetisation component $m_z$ along the out-of-plane direction.

To ensure consistency with the existing literature and for the sake of clarity, a few definitions are presented. When referring to non-collinear magnetic configuration, we indicate the following: (1) skyrmions are topological magnetic objects (see Fig. 1a) with a well-defined topological winding number and unique chirality that can arise in the presence of the DMI interaction. (2) SkBs are topological magnetic objects that are stabilized in the absence of the DMI interaction to satisfy the competition between dipolar interactions, magnetic anisotropy and Zeeman energy terms (see Fig. 1b). (3) magnetic bubbles are isolated, cylindrical regions of uniform magnetisation with trivial topology.

A material that has lately attracted significant attention for its complex magnetic phase diagram and non-trivial topological properties is ${\hbox {NdMn}_2\hbox {Ge}_2}$^28–30^. ${\hbox {NdMn}_2\hbox {Ge}_2}$ crystallizes in a tetragonal centrosymmetric structure (space group I4/${\text{mmm}}$). The structure can be described as composed of layers of alternating stacks of Nd, Mn, and Ge atomic layers along the *c*-axis. The Mn ions in $\hbox {NdMn}_{{2}}\hbox {Ge}_{{2}}$ host magnetic moments that order antiferromagnetically in the *a,b*-plane for temperatures below $T_N\sim 480~$K^30^. Upon further reduction of the temperature below $T_{c}\sim 340~$K, the moments cant towards the *c*-axis and, hence, develop a small ferromagnetic (FM) component along this direction (see Ref.^31^) in addition to the antiferromagnetic ordering present. This magnetic phase is stable down to $T\sim 240~$K, when a complex magnetic ordering pattern occurs, including a conical ordering at the Mn sublattice^28,31^.

Non-collinear magnetic structures arise from the sample’s magnetic anisotropy and can result in exotic behaviours such as a non-zero Hall voltage. Indeed, recently a large topological Hall effect (ToHE) was observed in $\hbox {NdMn}_{{2}}\hbox {Ge}_{{2}}$^28,30^. The ToHE is typically associated with the existence of the real-space topologically non-trivial spin textures that generate an emergent magnetic field and the associated extra Hall voltage. Through the use of Lorentz transmission electron microscopy (LTEM)^28,29^, it was shown that $\hbox {NdMn}_{{2}}\hbox {Ge}_{{2}}$ can host skyrmion-like magnetic structures at room temperature. These observations, including an unexpectedly large Hall resistivity $\rho _{H}\sim -2.05~\mu \Omega$ cm^28^, have attracted significant interest.

Despite this strong evidence for topologically non-trivial spin-textures in $\hbox {NdMn}_{{2}}\hbox {Ge}_{{2}}$, the mechanism for their stabilization is still unclear. One possible explanation is the formation of a Berry phase. DMI and spatial inversion asymmetry are more likely to give a large topological Hall effect, however, it is not necessary, as the presence of a non-collinear spin structure will result in a Berry phase, which will contribute to a topological Hall signal^32^.

To gain more insight into the complex magnetic configuration in $\hbox {NdMn}_{{2}}\hbox {Ge}_{{2}}$ and the mechanisms stabilizing topologically non-trivial spin-textures, we used several techniques. Using scanning transmission X-ray microscopy (STXM), we verified that a skyrmion bubble lattice (SkBL) exists at room temperature without an applied magnetic field and remains stable up to a higher transition temperature. Tests with an increasing out-of-plane magnetic field showed that the SkBL remains unchanged with tens of mT applied, and can partially recover if the field’s direction is reversed before magnetic saturation. Micromagnetic simulations, without a DMI term, matched the STXM experiments. Nitrogen-vacancy imaging provided quantitative information on the stray fields generated by the SkBL. Overall, our results confirm the stability of the SkBL in $\hbox {NdMn}_{{2}}\hbox {Ge}_{{2}}$ at room temperature without a magnetic field and their robustness against environmental changes. This suggests $\hbox {NdMn}_{{2}}\hbox {Ge}_{{2}}$ as an interesting candidate for possible spintronic applications.

## Results and discussion

Here, we aim to correlate our observations of the magnetic configuration using scanning transmission X-ray microscopy (STXM), with precise micromagnetic simulations. This allows us to rule out any previously undetected symmetry breaking and observe if the skyrmion core polarity switch would occur. STXM also allowed us to check the material’s suitability for 3D X-ray magnetic tomography by testing the strength of the X-ray magnetic circular dichroism (XMCD) at the Mn L edges.

For this, we first verified and maximised the XMCD signal for the terraced lamella (see Fig. S1b) using STXM (as described in the methods section) in the saturated state at room temperature. Next, we applied the field-cooling procedure “FP1” as described in the Methods section, to initialize the ${\hbox {NdMn}_2\hbox {Ge}_2}$ sample in the SkB phase.

**Fig. 2:**
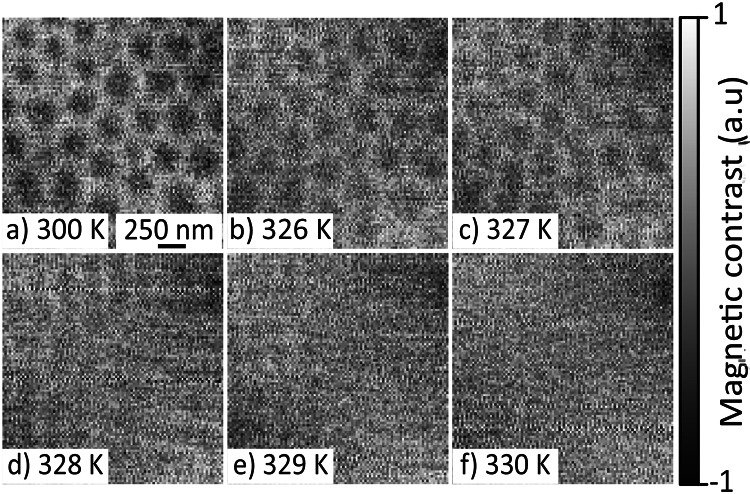
XMCD images acquired by STXM of the magnetic configuration’s temperature dependence from 300 K to the $T_c$ at 330 K. The field of view of the images is $2\times 2~\upmu \hbox {m}^{2}$ and they show the magnetic contrast observed with a single photon polarisation (circular right). (**a**) The skyrmion bubble lattice (SkBL) at 300 K after being initialised via the field cool. (**b**) At 326 K the SkBL is unchanged in shape and size. (**c**) The magnetic contrast starts to decrease at 327 K, but the SkBL is still stable. (**d**) At 328 K the SkBL has transitioned into a mixed helical state. (**e**) The helical phase still exists at 329 K, but the magnetic contrast is almost not visible. (**f**) The magnetic contrast shows that the sample is in its AFM regime at 330 K.

After applying FP1, we observed a regular skyrmion bubble lattice (SkBL), as shown in Fig. 2a, which remained stable after the removal of the applied magnetic field. Our measurements thereby confirm the creation of a metastable SkBL, as previously seen by Hou et al.^29^, in a single-crystal lamella of ${\hbox {NdMn}_2\hbox {Ge}_2}$.

After identifying the conditions required to generate the metastable SkBL at room temperature and zero field, we tested the stability of the SkBL against increasing temperature. For this, we heated the sample in zero-field, from room temperature towards $T_c$ in 1 K increments, taking an image at each step. Selected images from this temperature series are shown in Fig. 2 and show that the SkBL phase Fig. 2a–c, persists up to $T=327~$K, after which it transforms into a helical state Fig. 2d,e. This state was stable up to the $T_c$, at which no magnetic contrast was observed (Fig. 2f), as the sample transitioned into the AFM phase. Throughout the temperature range probed here (300-327 K), the SkBL is seen to be size and shape invariant, demonstrating its robustness against temperature through a technologically relevant temperature window. This set of measurements was of particular interest, as it allowed for the observation of topological melting from the SkBL configuration to an antiferromagnetic (AFM) state. In the AFM state, the magnetic moments align antiparallel to one another and lie within the layers of the Mn sublattice, giving null XMCD contrast.

**Fig. 3:**
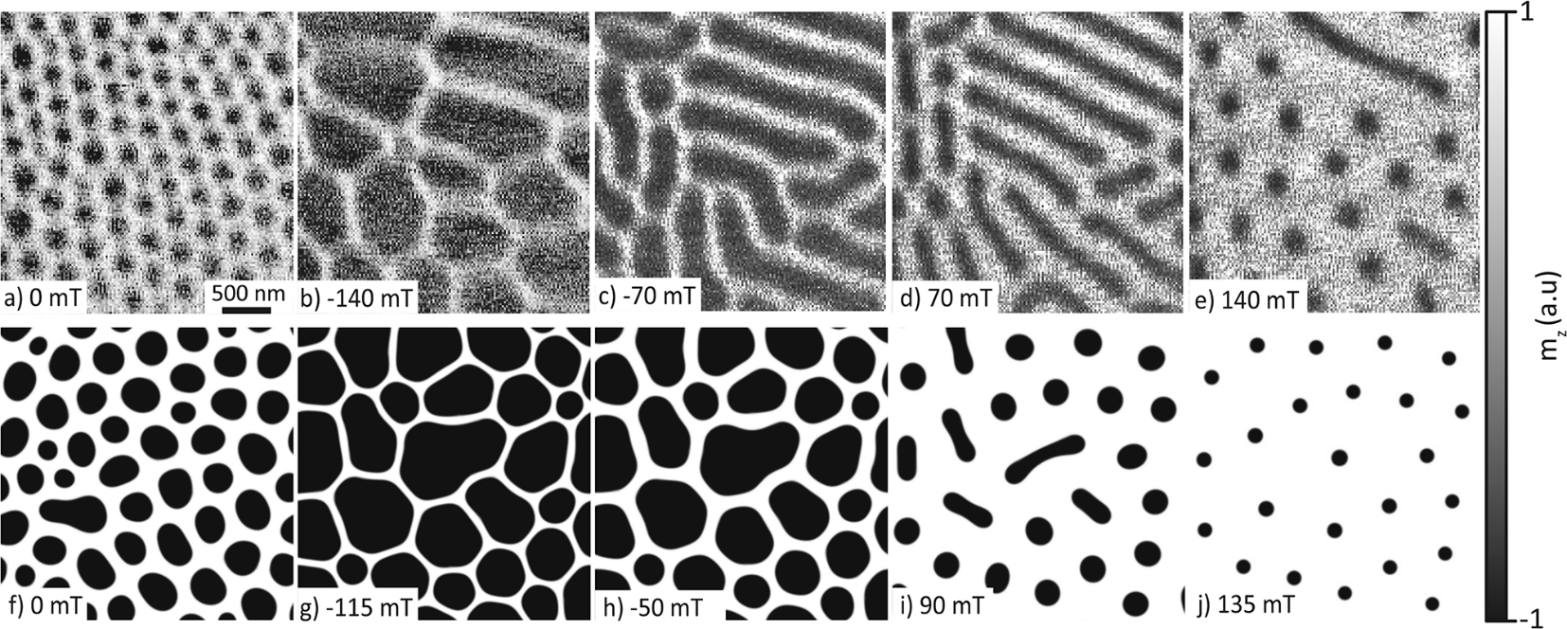
(**a**–**e**) The experimental XMCD field sweep data, where the field was swept in two directions. 50 mT FC, 0 mT image, − 140 mT $\rightarrow$ 140 mT in 10 mT increments (**f**–**j**) Simulations of the system without DMI, are similar to the experimental observations and show that the topological charge is mostly conserved throughout the field sweep. Shown here is the z-component of the magnetisation. The field of view for each XMCD and simulation image is 3 $\times$ 3 $\upmu \text {m}^{2}$.

Next, we explored the stability of the $\hbox {NdMn}_{{2}}\hbox {Ge}_{{2}}$ SkBL against magnetic field variations. Earlier studies have demonstrated skyrmion core polarity reversal under magnetic field sweeps, in systems that exhibit either bulk DMI^33^ or interfacial DMI^34^, while no reversal has been observed in a system with dipolar SkBLs^35^. In this latter work, it was proposed to use the observed magnetic field-induced collapsing dynamics and its associated skyrmion polarity change as potential fingerprints for identifying the type of skyrmions in magnetic multilayers. Therefore, core-polarity reversal could be taken as a good indicator of the presence or absence of DMI.

For this assessment, we first re-initialized the SkBL with the same magnetic field protocol FP1 and observed the evolution of the magnetic texture as a function of the magnetic field. The magnetic field was decreased in steps of 10 mT until a value of $-200~$mT, and images of the magnetic configurations were taken for each magnetic field value (see Fig. S2 in the Supplementary Information).

In a range of small negative magnetic fields (from $-10~$mT to $-90~$mT), metastable skyrmionic objects demonstrate coherent expansion of their cores, which is expected since the magnetic field was applied parallel to the magnetisation direction of the SkB core^36^. The expansion of the cores is homogeneous and results in a saturated state for the highest value of the magnetic field used. From these observations, we conclude that the skyrmion core reversal does not happen in ${\hbox {Nd}_2\hbox {Mn}_2\hbox {Ge}}$.

To test if the expansion of the skyrmion cores could be reversed and the starting SkBL could be recovered we devised a variation of the field protocol described above. After re-initializing the SkBL with FP1, the magnetic field was reduced from 50 mT until $-140~$mT, where SkBs were still observable (Fig. 3b). To avoid full saturation of the lamella and, thereby, the destruction of the SkB phase, the direction of the field sweep direction was reversed, and the field magnitude was reduced from $-140~$mT towards 0 mT. During this sequence the inflated skyrmion bubbles shrink (Fig. 3c), and subsequently elongate (Fig. 3d). After the field crosses zero and becomes positive, these elongated SkBs become thinner, eventually break apart, and again form an arrangement of SkB’s that we imaged at 140 mT (Fig. 3e). Compared to the SkBL initialised by our field cooling sequence (Fig. 3a), the SkBL resulting from this extended magnetic field history shows the same SkB polarity, but a significantly increased SkB spacing.

This demonstrates that in $\hbox {NdMn}_{{2}}\hbox {Ge}_{{2}}$, the deformation induced by the field sweep allows for the conservation of the topological charge to some extent, as previously seen in CoZnMn^33,37^.

To gather a more quantitative understanding of the mechanism driving the formation of a SkBL, we conducted micromagnetic simulations with field protocols and material parameters similar to the experiment and previous simulations ^29^ (see Table 1). In our simulations, we initialised the system in a random magnetic configuration in the presence of an out-of-plane magnetic field of 75 mT, in which the system was allowed to relax. This field value was chosen to best reproduce the experimental results but differs slightly from the field value of 50 mT employed in our experiment.

Subsequent to the in-field relaxation, the magnetic field was set to zero in the simulation and the system was allowed to relax again. The resulting magnetic state is shown in Fig. 3f and shows overall agreement with the experimental results, except for slightly enlarged SkB core sizes. From this simulation, to confirm the topological nature of the resulting SkBL, we extracted the winding number (*Q*) using the following equation^38^ and the x- and y-components of the reduced magnetisation ($\varvec{m}$) for the magnetic state of the simulation.

1 $$\begin{aligned} Q= \frac{1}{4\pi } \int \varvec{m} \cdot \left( \frac{\partial \varvec{m}}{\partial x} \times \frac{\partial \varvec{m}}{\partial y} \right) \textrm{d}x \textrm{d}y \end{aligned}$$

Use of Eq. (1) summing up all the magnetic configurations displayed in Fig. 3f (field cooled state) and Fig. 3j (final high field state) gives non-integer numbers for the total topological charge ($\hbox {Q}_T$ $=-35.3$ and $\hbox {Q}_T$ $= -25.5$, respectively), which is the result of using Eq. (1) and the discrete mesh used in the simulation^39^. The calculation was then repeated for volumes enclosing individual bubbles in Fig. 3j and we obtained an average value Q $= -0.98$ for all bubbles in this state, except for the bubble in the centre of the simulation, which has Q $= -1.94$. This is due to the collapsing of the elongated bubble state, which has Q $> 1$ before the collapse. When the applied magnetic field increases above 135 mT, this bubble has Q $\approx 1$, before the magnetic state becomes fully FM. This discrepancy in the calculated Q values is consistent with the one observed when calculating the topological skyrmion charge obtained by simulations on similar magnetic systems^39^. Taking note of these discrepancies we rounded our values for the total topological charge to the nearest integers, $\hbox {Q}_T=-35$ and $\hbox {Q}_T = -25$, respectively.

Based on the similarity between the objects in the simulations and experiment, we conclude, in agreement with early reports^28,29^, that the objects observed in the experiments are indeed SkBs.

Next, we use our simulations to identify the source of the SkB elongation seen in our magnetic field sweeps (Fig. 3c,d). For this, we first apply the field cooling procedure to initialise the system in a metastable SkBL at 0 mT (analogous to the FP1 protocol used during the experiments). Next, we let the SkB expand by decreasing the magnetic field from 0 to $-115~$mT in 5 mT steps, where the system was again allowed to relax at each step (Fig. 3g–j). This lower bound for the applied field was chosen to avoid full magnetic saturation of the system (and therefore the disappearance of the SkBs). Upon reaching $-115~$mT the field magnitude was increased to $+150~$mT, again in 5 mT steps, while letting the system relax at each point. An elongated skyrmion-like tube state was reached at 25 mT (not shown), after which the tubes started to shrink and break up into SkBs at 100 mT (Fig. 3i). At 135 mT we observe a SkBL that showed an increased lattice spacing compared to the initial state, exactly as observed in our experiment. Further increase of the field leads to a gradual disappearance of the SkBs and ultimately full saturation at 150 mT. Overall, we find good qualitative agreement between simulation and experiment regarding the behaviour of the SkBs.

**Table 1:** The parameters and physical constants values used for the simulations.

Simulations				
Parameter	No polarity switching	Polarity switching	NV comparison	Unit
World size	3072 $\times$ 3072 $\times$ 200	3072 $\times$ 3072 $\times$ 200	1980 $\times$ 2220 $\times$ 200	$\hbox {nm}^{3}$
Cell Size	3 $\times$ 3 $\times$ 5	3 $\times$ 3 $\times$ 200	3 $\times$ 3 $\times$ 5	$\hbox {nm}^{3}$
PBC	(2,2,0)	(2,2,0)	(2,2,0)	1
$\alpha$	1.0	1.0	1.0	1
$\text {D}_{\text {bulk}}$	0.0	1.0 $\times 10^{-3}$	0	J/$\hbox {m}^{2}$
$\text {A}_{\text {ex}}$	4 $\times$ 10^−^12	4 $\times$ 10^−^12	4 $\times$ 10^−^12	J/m
$\text {M}_{\text {sat}}$	220 $\times$ 10^3^	220 $\times$ 10^3^	220 $\times$ 10^3^	A/m
anisU	vector(0,0,1)	vector(0,0,1)	vector(0,0,1)	1
$\text {K}_{\text {u1}}$	5 $\times$ 10^4^	5 $\times$ 10^4^	5 $\times$ 10^4^	J/$\hbox {m}^{3}$

PBC is the periodic boundary conditions, $\alpha$ represents the Landau-Lifshitz damping constant, $\hbox {D}_{\text {bulk}}$ is the bulk DMI, $\hbox {A}_{\text {ex}}$ is the material’s exchange stiffness, the saturation magnetisation is $\hbox {M}_{\text {sat}}$, anisU is the uniaxial anisotropy direction and $\hbox {K}_{\text {u1}}$ is the 1st order uniaxial anisotropy constant.

**Fig. 4:**
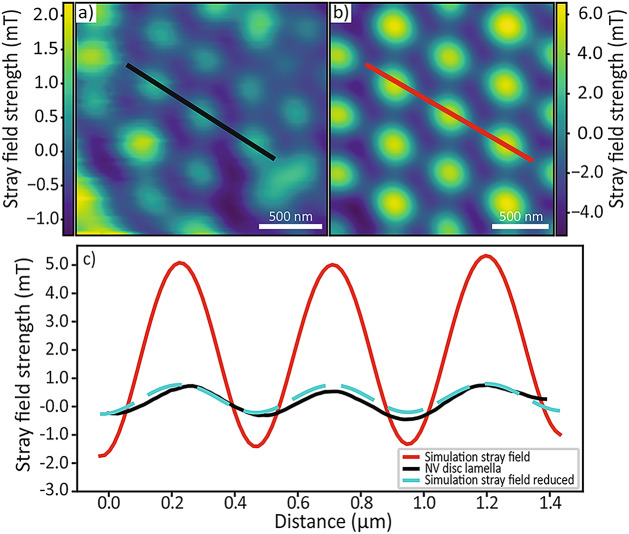
(**a**) Stray field map measured using NV magnetometry on a ${\hbox {NdMn}_2\hbox {Ge}_2}$ disk lamella, at a lift height of 300 nm. These stray field maps directly reflect the ferromagnetic moment of the canted antiferromagnetic sublattice. (**b**) A stray field map calculated from the disk lamella simulation at a lift height of 300 nm. (**c**) The black line profile shows the strength of the stray field for three skyrmion bubbles from the NV stray field map. A line profile for three skyrmion bubbles from simulations is plotted in red. Also plotted is the line profile (blue) calculated (using Eq. 2) from the simulation stray field data when accounting for dead layers of material and a different NV height.

To further underline the agreement between experiment and simulation and gain insights into $\hbox {NdMn}_2\hbox {Ge}_2$’s SkBL state, we used scanning NV magnetometry imaging to obtain a quantitative map of the magnetic stray field in close proximity to the sample.

Measurements were performed on a circular, $4~\mu$m diameter $\hbox {NdMn}_2\hbox {Ge}_2$ lamella of 190 nm thickness, that was FIB-cut from the same crystal as the lamella studied thus far [The $\hbox {NdMn}_2\hbox {Ge}_2$ lamella used for the X-ray experiments was inadvertently damaged during preliminary NV measuremnts. The stray field values obtained from this sample are comparable with the one obtained from the disk shape sample reported in the present manuscript.]. We initialised the SkBL as previously (FP1) and imaged the resulting SkBL under ambient conditions in a small bias field of 8.3 mT, to allow for quantitative NV magnetometry^40^. A representative result of our NV measurements is shown in Fig. 4a, and shows a stray-field image of a highly regular, triangular $\hbox {NdMn}_2\hbox {Ge}_2$ SkBL, with a $\sim 490~$nm SkB spacing, largely consistent with our previous STXM findings (Fig. 2a).

This image directly yields a quantitative estimate of the stray magnetic field strength at the scan-height $h_{NV}\approx 300~$nm of the NV sensor. To benchmark this quantitative measurement against our model, we conducted a second set of simulations, using the same material parameters as earlier (see Tab. 1), but initialized the system with a Bloch skyrmion lattice that we subsequently relaxed. With this initialisation, it was possible to obtain a SkBL configuration closely resembling the NV experimental results (see Fig. 4b). The stray field was then computed up to a distance of 500 nm away from the sample surface and projected onto the known direction of the NV spin quantization axis ($55^{\circ }\pm 4^{\circ }$ from the sample normal, azimuthal angle $30^{\circ }\pm 4^{\circ }$) to reproduce the anticipated NV magnetometry image from the simulations. Figure 4b shows an example of such a simulated stray field map, evaluated at $h_{NV}=300~$nm. Our comparison shows that the measured field strength is $\sim 7\times$ weaker than the one predicted from our simulations, as seen in the linecuts in Fig. 4c.

This difference in stray field magnitude may result from several factors, which we discuss in the following:

First, given that the stray field strength scales directly with the magnetisation strength $M_{sat}$, the observed discrepancy could be explained by the choice of the value of $M_{sat}$ in our simulations. There, we initially set $M_{sat}$ to the full sublattice magnetisation of $\hbox {NdMn}_2\hbox {Ge}_2$, in accordance with past works^29^. However, previous studies^28,31^ indicate that for 240 K$<T<340~$K, a canting of the sublattice magnetisation occurs as the out-of-plane ferromagnetic state transitions to an in-plane antiferromagnetic state. At $T=285~$K, a canting angle of $58^\circ$ is reported^31^, which would reduce the ferromagnetic moment of $\hbox {NdMn}_2\hbox {Ge}_2$ by a factor of two over the value we chose for $T=300~$K, where our experiments were conducted.

Second, due to manufacturing by FIB there is a surface layer where the material’s crystalline structure is damaged. The thickness of these “dead-layers” is on the order of a few tens of nanometers on each side of the lamellae^41^ and would further reduce the stray field strength from the expected value. Based on standard theory for field propagation away from thin magnetic films^42^, these factors can be used to estimate the resulting reduction in stray field magnitude through the “thickness loss factor”.

2 $$\begin{aligned} B \propto \exp \left( -\frac{2\pi d_\text {top}}{p_\text {skyr}}\right) - \exp \left( -\frac{ 2 \pi d_{\text {bot}}}{p_\text {skyr}}\right) , \end{aligned}$$

with $d_\text {top} = h_{NV} + t_d$ and $d_{\text {bot}} = h_{NV} - t_d + t$, where *t*, $t_d$ are the thicknesses of the magnetically ordered part of the $\hbox {NdMn}_2\hbox {Ge}_2$ lamella and dead layer, respectively, and $p_\text {skyr}$ is the periodicity of the SkBL.

Considering all these factors, the observed discrepancy in stray field strength could be explained by a realistic set of parameters, such as a canting angle of $68^\circ$, $h_{NV}=340~$nm and $t_d=35~$nm see blue dashed curve in Fig. 4c. While it is beyond the scope of the current manuscript to disentangle the different possible contributions, the quantification of the reduced stray field values may provide valuable information in future studies on the canting angle or non-magnetic dead layers.

## Conclusion

In summary, we have scrutinised the nature of skyrmionic objects in $\hbox {NdMn}_{{2}}\hbox {Ge}_{{2}}$ using micromagnetic simulations, scanning transmission X-ray microscopy imaging and nitrogen-vacancy magnetometry. As already reported, with an appropriate field cooling protocol, the system is capable of hosting a metastable SkBL at room temperature, in zero magnetic field. This SkBL is robust to changes in temperature from room temperature up until the sample’s $T_c$. We further demonstrate that, upon the application of an external magnetic field it is not possible to reverse the SkBs’ core polarity. Interestingly when the field is taken from 50 mT to $-200~$mT and then ramped to 200 mT, the SkBL deforms into FM domains and then restores into a SkBL with a reduced number of SkBs. All of our observations could be reproduced by micromagnetic simulations incorporating exchange interaction as well as magnetic anisotropy. The simulations revealed that including an extra DMI term makes it possible to reverse the SkBL polarity. This suggests that the inability to reverse the skyrmion’s core polarity is due to the absence of DMI in $\hbox {NdMn}_{{2}}\hbox {Ge}_{{2}}$. Finally, our NV magnetometry measurements provide a quantitative assessment of the magnetic stray field of the sample at a height of 300 nm above the sample surface, suggesting that the micromagnetic simulations overestimated it by a factor of 7. This can be accounted for by considering the dead layers arising from the sample preparation, a slightly higher NV lift height, and the canting of the sublattice magnetisation.

## Methods

### Sample growth

Single crystals of $\hbox {NdMn}_{{2}}\hbox {Ge}_{{2}}$ were synthesized using the Sn-flux method. A mixture of elements with an atomic ratio of Nd:Mn:Ge:Sn=1:2:2:10 was sealed in an evacuated quartz tube and heated to $1000~^\circ$C over 20 h (heating rate $50~^\circ$C/h) and held at that temperature for 10 h. The furnace was then cooled to $500~^\circ$C over 100 h (cooling rate $5~^\circ$C/h). After this, the sample was quenched to room temperature, and any remaining flux was decanted using a centrifuge.

### Lamella preparation by FIB

We performed the preparation of the lamellae using a gallium Focused Ion Beam (FIB) Scanning Electron Microscope (SEM), a Thermo Scientific Helios 5 UX DualBeam, equipped with a MultiChem gas injection system and an EasyLift micromanipulator needle with a motorized rotation axis. The procedure employed is described in Ref.^43^. A single crystal was oriented, embedded, and polished so that the extracted lamella was (001)-oriented. 30 kV Ga ions were used to shape and thin down the lamellae on the manipulator needle. Before reaching the target thickness the ion energy was dropped to 5 kV to ensure high crystallinity of the samples. The thickness of the lamella was determined by FIB imaging top-down as well as the electron energy required for seeing transparency in SEM imaging. The terraced lamella has three $4\times 6~\mu$m sized windows with a thickness of 189 nm $\pm 10~$nm, for the first, and 216 nm $\pm 10~$nm for the second and third window (see electron microscopy image in the inset to Fig. S1b). To shape a disk, the lamella is thinned to target thickness all over and rotated such that the FIB incidence is practically normal to the lamella plane. A small bridge to the disk is left. Finally, the lamella or disk is mounted onto a 200  nm thick SiNx (silicon-rich nitride) membrane, which acts as an X-ray transparent sample carrier. This membrane additionally contained electrical leads for sample heating, consisting of suitably shaped, 60 nm thick Pt stripes (see inset of Fig. S1b), fabricated using electron beam lithography. After the lamella or disk is in the required position it is fixed by depositing carbon using a 5 kV Ga ion beam. To release the lamella or disk from the manipulator, a trench is milled through the lamella rather than through the manipulator needle to avoid re-deposition of tungsten on the lamella.

### Skyrmion bubble lattice stabilization protocol

Before each measurement in the experiments (STXM and NV magnetometry), the magnetic state of the lamella was reset to a skyrmion bubble lattice. To achieve this the following field cooling procedure (identified as FP1) was used. First, the lamella was heated above $\hbox {T}_{{c}}$ via the application of a mA current through the Pt heater strips on the membrane at a rate of $1.6~ \Omega \hbox {K}^{-1}$. This current was provided and controlled by a Keithley 2400 source meter and was calibrated using another heater chip. A small magnetic field of 50 mT was applied out-of-plane (perpendicular to the sample’s surface) while the sample was still being heated. This arrangement was maintained for one minute, after which the current was gradually decreased and then turned off. The sample was allowed to cool for an additional 30 seconds to reach room temperature. Finally, the magnetic field was removed and the sample was imaged.

### Scanning transmission X-ray microscopy

Scanning transmission X-ray microscopy (STXM) was used to obtain images of the sample’s magnetic configuration leveraging the X-ray Magnetic Circular Dichroism (XMCD), present at the Mn $\hbox {L}_{{3}}$ edge (641 eV). The STXM measurements were performed at the PolLux beamline of the Swiss Light Source (SLS)^44^ in a 2D setup configuration. The X-ray beam was focused using a Fresnel zone plate with an outermost zone width of 25 nm and whose first diffraction order was selected with an order sorting aperture. A typical STXM image is composed of $100\times 100~$pixels with an acquisition time of 30 ms per pixel. A computer-controlled motorised setup with a permanent magnet was used to modulate the strength of the applied out-of-plane magnetic field. The electrical leads on the SiNx membrane allowed for in-situ heating of the sample above $T_c$ via the application of currents in the mA range.

### Nitrogen-vacancy imaging

NV magnetometry was used to obtain quantitative stray field maps in an imaging plane close to the sample surface. NV magnetometry utilizes the electronic spin of NV centres in diamond as highly sensitive, local magnetic field sensors, capable of detecting magnetic fields with nanoscale spatial resolution^40^. In this, the NV spin is sensitive to magnetic fields projected along its spin quantisation axis, which in the implementation used here^45^ is tilted from the sample normal by an angle of $55^{\circ }\pm 4^{\circ }$. This value coincides with the expected axis orientation from the (001)-diamond cut. Its magnitude and the value of the inplane orientation of $30^{\circ }\pm 4^{\circ }$ have been determined from a precise magnetic field alignment and additional fitting of stray fields from reference samples.

The standoff distance in AFM contact has been determined to 50±5 nm based on stray field measurements on reference samples, while all piezo calibrations were performed using laser-interferometry and commercially available height calibration samples. The total resulting lift height is estimated to be 300 nm. This value is larger than the initially targeted 260 nm lift, but appears very plausible due to possible tip contamination and thermal drifts that result in larger distances and smaller stray field strength, given the large discrepancy in the expected and measured stray fields.

### Micromagnetic simulations

Several micromagnetics simulations were conducted using the software MuMax3^46^. Two of the simulations had a world size of $3072\times 3072\times 200~\hbox {nm}^{3}$, representing the area of the sample that was measured with STXM. The other simulation had a world size of $1980\times 2220\times 200~\hbox {nm}^{3}$ which represents the area measured with NV magnetometry. All simulations had cell size of $3\times 3\times 5~\hbox {nm}^{3}$, with lateral dimensions well below the exchange length $l_{ex}=\sqrt{A_{ex}/M_s^2}$, where $A_{ex}$ is the exchange constant and $M_s$ the saturation magnetisation. We used periodic boundary conditions (PBC) set to (2,2,0), representing two repetitions of the demagnetisation field in the x- and y-axis. This ensured that any interactions caused through the demagnetising field from the material surrounding the measured window were captured. The parameters used in the simulations are shown in Table 1, where the values for $\hbox {A}_{\text {ex}}$, $\hbox {M}_{\text {sat}}$ and $\hbox {K}_{\text {u1}}$ (the uniaxial magnetic anisotropy constant) are taken from Ref.^29^.

## Supplementary Information

Supplementary Information.

## Data Availability

The datasets generated and/or analysed during the current study are available in the ZENODO repository, 10.5281/zenodo.11072737.

## References

[1] Skyrme, T. H. A unified field theory of mesons and baryons. *Nucl. Phys.* **31**, 556. 10.1016/0029-5582(62)90775-7 (1962).

[2] Bogdanov, A. N. & Yablonskii, D. A. Thermodynamically stable vortices in magnetically ordered crystals. *Sov. Phys. JETP* **68**, 101 (1989).

[3] Fert, A., Cros, V. & Sampaio, J. Skyrmions on the track. *Nat. Nanotechnol.* **8**, 152. 10.1038/nnano.2013.29 (2013). 23459548 10.1038/nnano.2013.29

[4] Jonietz, F. et al. Spin transfer torques in MnSi at ultralow current densities. *Science* **330**, 1648. 10.1126/science.1195709 (2010). 21164010 10.1126/science.1195709

[5] Yu, X. Z. et al. Skyrmion flow near room temperature in an ultralow current density. *Nat. Commun.* **3**, 988. 10.1038/ncomms1990 (2012). 22871807 10.1038/ncomms1990

[6] Iwasaki, J., Mochizuki, M. & Nagaosa, N. Current-induced skyrmion dynamics in constricted geometries. *Nat. Nanotechnol.* **8**, 742. 10.1038/nnano.2013.176 (2013). 24013132 10.1038/nnano.2013.176

[7] Mühlbauer, S. et al. Skyrmion lattice in a chiral magnet. *Science* **323**, 915. 10.1126/science.1166767 (2009). 19213914 10.1126/science.1166767

[8] Yu, X. Z. et al. Near room-temperature formation of a skyrmion crystal in thin-films of the helimagnet FeGe. *Nat. Mater.* **10**, 106. 10.1038/nmat2916 (2011). 21131963 10.1038/nmat2916

[9] Seki, S., Yu, X. Z., Ishiwata, S. & Tokura, Y. Observation of skyrmions in a multiferroic material. *Science* **336**, 198. 10.1126/science.1214143 (2012). 22499941 10.1126/science.1214143

[10] Tokunaga, Y. et al. A new class of chiral materials hosting magnetic skyrmions beyond room temperature. *Nat. Commun.* **6**, 7638. 10.1038/ncomms8638 (2015). 26134284 10.1038/ncomms8638 PMC4506512

[11] Kézsmárki, I. et al. Néel-type skyrmion lattice with confined orientation in the polar magnetic semiconductor GaV_4_S_8_. *Nat. Mater.* **14**, 1116. 10.1038/nmat4402 (2015). 26343913 10.1038/nmat4402

[12] Kurumaji, T. et al. Néel-type skyrmion lattice in the tetragonal polar magnet VOSe_2_O_5_. *Phys. Rev. Lett.* **119**, 237201. 10.1103/PhysRevLett.119.237201 (2017). 29286691 10.1103/PhysRevLett.119.237201

[13] Dzyaloshinsky, I. A thermodynamic theory of “weak’’ ferromagnetism of antiferromagnetics. *J. Phys. Chem. Solids* **4**, 241. 10.1002/pssb.2220460236 (1958).

[14] Moriya, T. Anisotropic superexchange interaction and weak ferromagnetism. *Phys. Rev.* **120**, 91. 10.1103/PhysRev.120.91 (1960).

[15] Adams, T. et al. Long-wavelength helimagnetic order and skyrmion lattice phase in Cu_2_OSeO_3_. *Phys. Rev. Lett.* **108**, 237204. 10.1103/PhysRevLett.108.237204 (2012). 23003986 10.1103/PhysRevLett.108.237204

[16] Moreau-Luchaire, C. et al. Additive interfacial chiral interaction in multilayers for stabilization of small individual skyrmions at room temperature. *Nat. Nanotechnol.* **11**, 444. 10.1038/nnano.2015.313 (2016). 26780660 10.1038/nnano.2015.313

[17] Yang, H., Thiaville, A., Rohart, S., Fert, A. & Chshiev, M. Anatomy of Dzyaloshinskii-Moriya Interaction at Co/Pt interfaces. *Phys. Rev. Lett.* **115**, 267210. 10.1103/PhysRevLett.115.267210 (2015). 26765026 10.1103/PhysRevLett.115.267210

[18] Yu, X. Z. et al. Real-space observation of a two-dimensional skyrmion crystal. *Nature* **465**, 901. 10.1038/nature09124 (2010). 20559382 10.1038/nature09124

[19] Tokura, Y. & Kanazawa, N. Magnetic skyrmion materials. *Chem. Rev.* **121**, 2857. 10.1021/acs.chemrev.0c00297 (2021). 33164494 10.1021/acs.chemrev.0c00297

[20] Karube, K. et al. Skyrmion formation in a bulk chiral magnet at zero magnetic field and above room temperature. *Phys. Rev. Mater.* **1**, 074405. 10.1103/PhysRevMaterials.1.074405 (2017).

[21] Peng, L. et al. Formation and control of zero-field antiskyrmions in confining geometries. *Adv. Sci.* **9**(28), 2202950. 10.1002/advs.202202950 (2022). 10.1002/advs.202202950 PMC9534945 35978271

[22] Fert, A., Reyren, N. & Cros, V. Magnetic skyrmions: Advances in physics and potential applications. *Nat. Rev. Mater.* **2**, 17031. 10.1038/natrevmats.2017.31 (2017).

[23] Wang, W. et al. A centrosymmetric hexagonal magnet with superstable biskyrmion magnetic nanodomains in a wide temperature range of 100k–340K. *Adv. Mater.* **28**, 6887. 10.1002/adma.201600889 (2016). 27192410 10.1002/adma.201600889

[24] Montoya, S. A. et al. Tailoring magnetic energies to form dipole skyrmions and skyrmion lattices. *Phys. Rev. B* **95**, 024415. 10.1103/PhysRevB.95.024415 (2017).

[25] Hou, Z. et al. Observation of various and spontaneous magnetic skyrmionic bubbles at room temperature in a frustrated kagome magnet with uniaxial magnetic anisotropy. *Adv. Mater.* **29**, 1701144. 10.1002/adma.201701144 (2017). 10.1002/adma.201701144 28589629

[26] Yu, X. Z. et al. Biskyrmion states and their current-driven motion in a layered manganite. *Nat. Commun.* **5**, 3198. 10.1038/ncomms4198 (2014). 24469318 10.1038/ncomms4198

[27] Chakrabartty, D., Jamaluddin, S., Manna, S. K. & Nayak, A. K. Tunable room temperature magnetic skyrmions in centrosymmetric kagome magnet Mn_4_Ga_2_Sn. *Commun. Phys.* **5**, 189. 10.1038/s42005-022-00971-7 (2022).

[28] Wang, S. et al. Giant topological hall effect and superstable spontaneous skyrmions below 330 K in a centrosymmetric complex noncollinear ferromagnet NdMn_2_Ge_2_. *ACS Appl. Mater. Interfaces* **12**, 24125. 10.1021/acsami.0c04632 (2020). 32363848 10.1021/acsami.0c04632

[29] Hou, Z. et al. Emergence of room temperature stable skyrmionic bubbles in the rare earth based REMn_2_Ge_2_ (RE = Ce, Pr, and Nd) magnets. *Mater. Today Phys.* **17**, 100341. 10.1016/j.mtphys.2021.100341 (2021).

[30] Zheng, X. et al. Giant topological hall effect around room temperature in noncollinear ferromagnet NdMn_2_Ge_2_ single crystal. *Appl. Phys. Lett.* **118**, 072402. 10.1063/5.0033379 (2021).

[31] Welter, R., Venturini, G., Ressouche, E. & Malaman, B. Neutron diffraction study of CeMn_2_Ge_2_, PrMn_2_Ge_2_ and NdMn_2_Ge_2_: Evidence of dominant antiferromagnetic components within the (001) Mn planes in ferromagnetic ThCr_2_Si_2_-type manganese ternary compounds. *J. Alloy. Compd.* **218**, 204. 10.1016/0925-8388(94)01378-0 (1995).

[32] Nagaosa, N. & Tokura, Y. Emergent electromagnetism in solids. *Phys. Scr.* **2012**, 014020. 10.1088/0031-8949/2012/T146/014020 (2012).

[33] Ukleev, V. et al. Topological melting of the metastable skyrmion lattice in the chiral magnet Co_9_Zn_9_Mn_2_. *Adv. Quantum Technol.* **5**, 2200066. 10.1002/qute.202200066 (2022).

[34] Tomasello, R. et al. Field-driven collapsing dynamics of skyrmions in magnetic multilayers. *Phys. Rev. B* **107**, 184416. 10.1103/PhysRevB.107.184416 (2023).

[35] Tang, J. et al. Skyrmion-bubble bundles in an X-Type Sr_2_Co_2_Fe_28_O_46_ hexaferrite above room temperature. *Adv. Mater.* **35**, 2306117. 10.1002/adma.202306117 (2023). 10.1002/adma.202306117 37668003

[36] Pierobon, L., Moutafis, C., Li, Y., Löffler, J. F. & Charilaou, M. Collective antiskyrmion-mediated phase transition and defect-induced melting in chiral magnetic films. *Sci. Rep.* **8**, 1. 10.1038/s41598-018-34526-0 (2018). 30420698 10.1038/s41598-018-34526-0 PMC6232090

[37] Morikawa, D. et al. Deformation of topologically-protected supercooled skyrmions in a thin plate of chiral magnet Co_8_Zn_8_Mn_4_. *Nano Lett.* **17**, 1637. 10.1021/acs.nanolett.6b04821 (2017). 28135106 10.1021/acs.nanolett.6b04821

[38] Heinze, S. et al. Spontaneous atomic-scale magnetic skyrmion lattice in two dimensions. *Nat. Phys.* **7**, 713. 10.1038/nphys2045 (2011).

[39] Kim, J.-V. & Mulkers, J. On quantifying the topological charge in micromagnetics using a lattice-based approach. *IOP SciNotes* **1**, 025211. 10.1088/2633-1357/abad0c (2020).

[40] Rondin, L. et al. Stray-field imaging of magnetic vortices with a single diamond spin. *Nat. Commun.* **4**, 2279. 10.1038/ncomms3279 (2013). 23900221 10.1038/ncomms3279

[41] Giannuzzi, L. & Stevie, F. A review of focused ion beam milling techniques for TEM specimen preparation. *Micron* **30**, 197. 10.1016/S0968-4328(99)00005-0 (1999).

[42] Meyer, E., Hug, H. J. & Bennewitz, R. *Scanning Probe Microscopy* (Springer, 2004). 10.1007/978-3-662-09801-1.

[43] Thermoscientific, FIB TEM Sample Preparation for in situ heating in TEM (2017). https://assets.thermofisher.com/TFS-Assets/MSD/Application-Notes/fib-tem-sample-preparation-in-situ-heating-tem-application-note.pdf

[44] Raabe, J. et al. Pollux: A new facility for soft X-ray spectromicroscopy at the swiss light source. *Rev. Sci. Instrum.* **79**, 113704. 10.1063/1.3021472 (2008). 19045892 10.1063/1.3021472

[45] Hedrich, N., Rohner, D., Batzer, M., Maletinsky, P. & Shields, B. J. Parabolic diamond scanning probes for single-spin magnetic field imaging. *Phys. Rev. Appl.* **14**, 064007. 10.1103/PhysRevApplied.14.064007 (2020).

[46] Vansteenkiste, A. et al. The design and verification of MuMax3. *AIP Adv.* **4**, 107133. 10.1063/1.4899186 (2014).

