# Supplementary material for: Investigating skyrmion stability and core polarity reversal in NdMn2Ge2
Source: Sci Rep. 2025 Jan 2;15:461. doi: 10.1038/s41598-024-82114-2 (PMC11697451; doi:10.1038/s41598-024-82114-2)
Supplement: Supplementary file 1 — Supplementary Information. [file 41598_2024_82114_MOESM1_ESM.pdf]

Investigating Skyrmion stability and core polarity reversal in $NdMn_2Ge_2$ Supplementary Information

Samuel K. Treves[1,2,3], Victor Ukleev[3,4], Andreas Apseros[2,3], Jamie Robert Massey[2,3], Kai Wagner[1], Paul Lehmann[1], Aki Kitaori[5,6], Naoya Kanazawa[7], Jeffrey A. Brock[2,3], Simone Finizio[8], Joakim Reuteler[9], Yoshinori Tokura[5,10,11], Patrick Maletinsky[1], and Valerio Scagnoli[2,3]*

[1] *Department of Physics, University of Basel, 4056 Basel, Switzerland*
[2] *Laboratory for Mesoscopic Systems, Department of Materials, ETH Zurich, 8093 Zurich, Switzerland*
[3] *PSI Center for Neutron and Muon Sciences, 5232 Villigen PSI, Switzerland*
[4] *Helmholtz-Zentrum Berlin für Materialien und Energie, D-14109 Berlin, Germany*
[5] *Department of Applied Physics, University of Tokyo, Tokyo 113-8656, Japan*
[6] *Institute of Engineering Innovation, The University of Tokyo, Tokyo 113-0032, Japan*
[7] *Institute of Industrial Science, The University of Tokyo, 4-6-1 Komaba Meguro-ku, Tokyo 153-8505, Japan*
[8] *PSI Center for Photon Science, 5232 Villigen PSI, Switzerland*
[9] *ScopeM, ETH Zurich, 8093 Zurich, Switzerland*
[10] *RIKEN Center for Emergent Matter Science (CEMS), Wako 351-0198, Japan and*
[11] *Tokyo College, University of Tokyo, Tokyo 113-8656, Japan*

SAMPLE CHARACTERIZATION

A crystal was selected from a group of crystals that were grown together. Its crystalline structure was characterised using a Bruker D8 Discover diffractometer with monochromated Cu–K_α radiation (λ=1.54 Å). This produced the 2θ-ω X-ray diffraction (XRD) pattern presented in Fig. S1a. The presence of the (002), (004) and (008) reflections at the expected positions, dictated by the c-axis lattice spacing, indicate that the crystal has the correct chemical composition and lattice spacing, as reported in [1]. The temperature dependence of the sample magnetisation was determined using a Quantum Design magnetic property measurement system (MPMS3) superconducting quantum interference device vibrating sample magnetometer (Fig. S1b). These data show the occurrence of all expected magnetic phase transitions for $NdMn_2Ge_2$ [1] and thereby further confirm the high quality of the sample. In particular, the transition temperature to the non-collinear state with a ferromagnetic contribution was determined to $T_c \sim 335$ K.

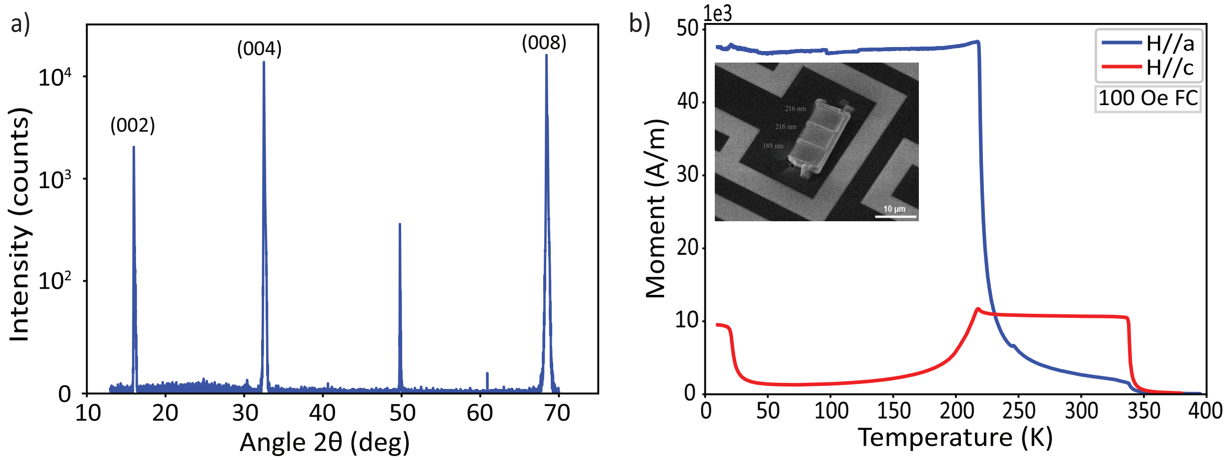

FIG. S1. $NdMn_2Ge_2$ sample characterisation. a) $NdMn_2Ge_2$ XRD measurements with Bragg peaks indexed. The shape and the spacing of the (002), (004), (008) reflections indicate the good quality of the crystal structure. b) Temperature dependence of the sample magnetisation recorded for decreasing temperature in a constant bias magnetic field of 10 mT applied along the a and c axes (blue and red, respectively). The inset shows a scanning electron microscopy image of the terraced $NdMn_2Ge_2$ lamella used in the x-ray experiments, placed on a holder equipped with a Pt wire-circuit for sample heating. The scale bar is 10 μm.

MAGNETIC FIELD SWEEP

During the x-ray imaging experiment, two magnetic field sweeps were performed. One of these had an out-of-plane (OOP) magnetic field applied parallel to the skyrmion bubble (SkB) cores (negative field sweep), and the other was applied anti-parallel to the SkB cores (positive field sweep). Before either procedure was conducted the system's magnetic state was reset with the field-cooling protocol (FP1) mentioned in the main paper. The subsequent measurements were then conducted at room temperature.

The results for the negative field sweep are seen in Fig. S2. After conducting FP1, we start with a skyrmion lattice. This magnetic state is seen in Fig. S2a, with a magnetic field of 50 mT being applied. The magnetic field was then reduced in 10 mT steps until 0 mT. After measuring at 0 mT the magnetic field was applied in the same direction as the magnetisation of the skyrmion bubble cores, and steps of -10 mT were made until the applied field reached -200 mT. In a range of small magnetic fields (-10 to -90 mT), the metastable skyrmion bubbles demonstrate a coherent expansion of their cores (Fig. S2b-c). Further core expansions simultaneously occur whilst neighbouring cores merge until magnetic fields of -120 mT. Further increments of the field magnitude, from -130 mT onwards, lead to more expansion of these skyrmion bubbles (Fig. S2d-e) and eventually their collapse at the critical field of -160 mT (Fig. S2f).

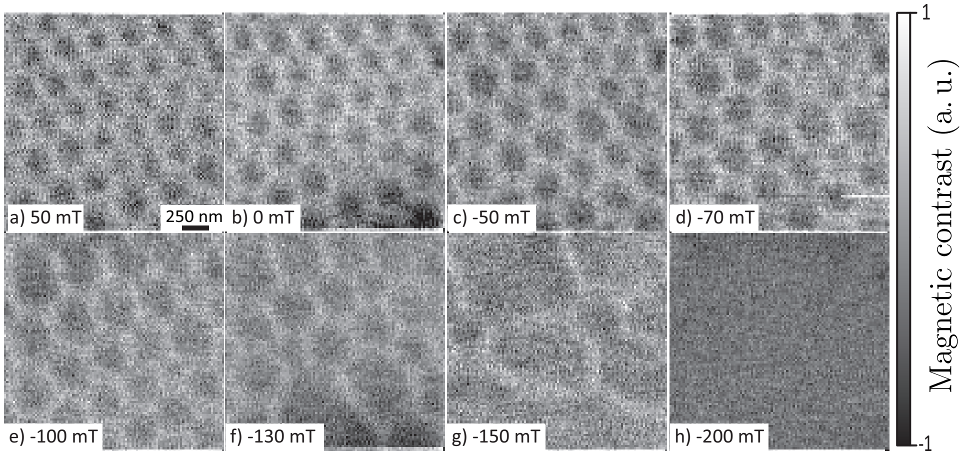

FIG. S2. X-ray projections with circular right polarisation for the experimental negative field sweep. a) Field cooled state, with the OOP magnetic field still applied. b-f) Selected projections from the magnetic field sweep, projections were taken every 10 mT. The field of view for each projection is 2×2 μm

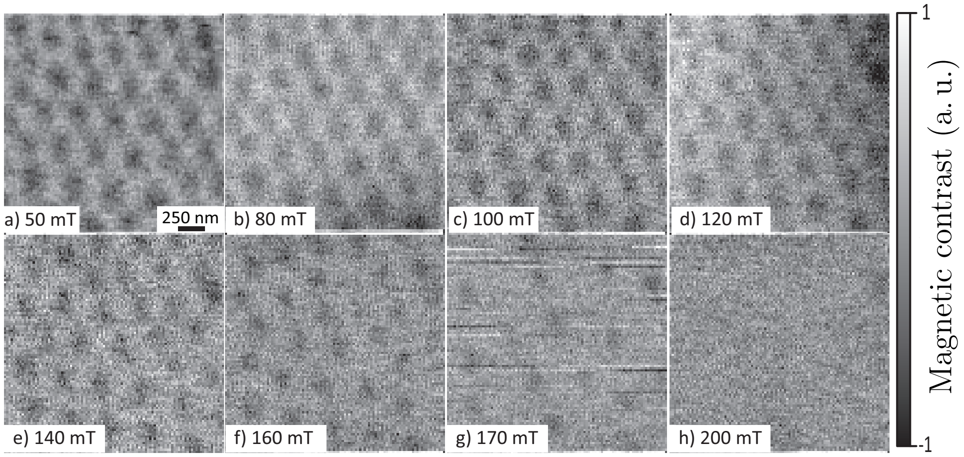

FIG. S3. X-ray projections with circular right polarisation for the experimental positive field sweep. a) Field cooled state with a magnetic field of 50 mT still applied. b-f) Selected projections from the magnetic field sweep, which had a maximum magnetic field of 200 mT, and 10 mT field increments. The field of view for each projection is 2×2 μm

As no core polarity switching was observed with the parallel field procedure, we repeated the procedure with the direction of the applied magnetic field being antiparallel to the magnetisation direction of the cores of the skyrmion-like objects. Starting from 50 mT (Fig. S3a), the applied field was stepped up to 200 mT in 10 mT increments. We observe that the lattice configuration remains mostly unchanged (Fig. S3b-d), but begins to break into individual skyrmion bubbles at 170 mT (Fig. S3e). With the application of magnetic fields up to 200 mT, the resulting final state of the system is a homogeneous fully ferromagnetic state. Similar behaviour has been demonstrated at 6 K in the quenched skyrmion lattice state in FeGe [2].

SIMULATIONS WITH BULK DMI TERM

As mentioned in the main paper $NdMn_2Ge_2$ hosts a large topological Hall effect. To exclude the possibility of this being caused by the Dzyaloshinskii–Moriya interaction (DMI), a further simulation was conducted with a non-zero bulk DMI parameter. The protocol for this simulation was a magnetic field sweep, similar to our initial experimental procedure. First, we imitate a field cool of the system from an initial random magnetic state, using an OOP magnetic field of 400 mT and allowing the system to relax. Once the system had fully relaxed the applied field was switched off, resulting in a stable skyrmion lattice (Fig. S4b). The OOP magnetic field was then applied parallel to the skyrmion cores, starting from 0 T and swept until -1 T. The field was increased in -10 mT increments, and the system was allowed to relax at each field step. A reversal of the skyrmion core polarity is observed in this case, which is distinct from what was observed experimentally.

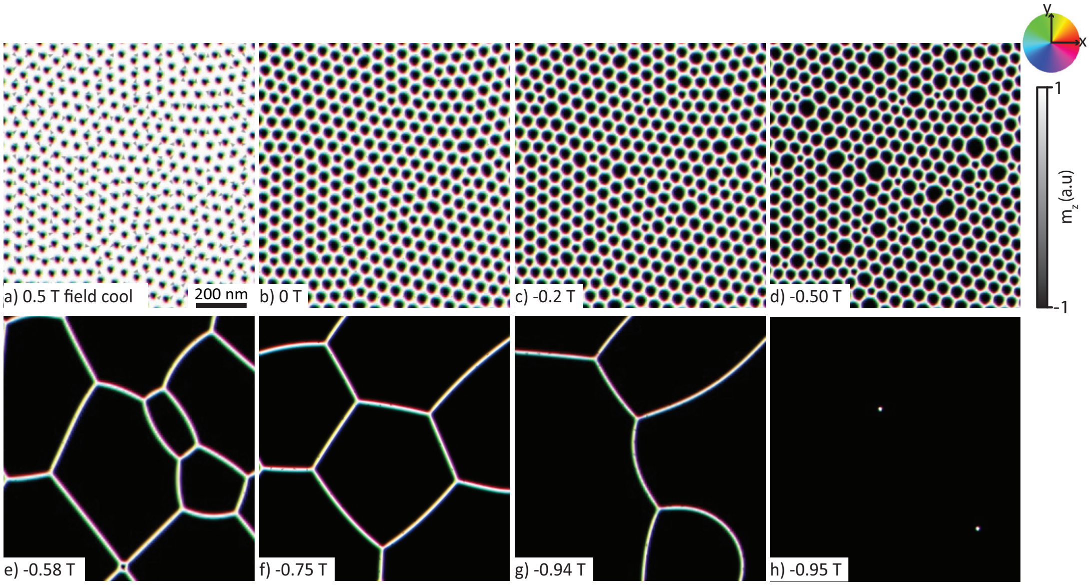

FIG. S4. Selected relaxed magnetic states with a cropped $1 \times 1\ \mu m^2$ field of view, from the micromagnetic simulation of the modified $NdMn_2Ge_2$ system which includes a large bulk DMI. Starting from a field cooled state (a), initial increases in the strength of the applied magnetic field parallel to the skyrmion core's magnetisation result in the merging of the skyrmions into magnetic domains (b-e). These domains continue to merge with increasing magnetic field (f-g), and eventually collapse into skyrmions (h). The magnetisation of the skyrmion cores is the opposite polarity of those in the starting skyrmion lattice.

[1] S. Wang, Q. Zeng, D. Liu, H. Zhang, L. Ma, G. Xu, Y. Liang, Z. Zhang, H. Wu, R. Che, X. Han, and Q. Huang, Giant Topological Hall Effect and Superstable Spontaneous Skyrmions below 330 K in a Centrosymmetric Complex Noncollinear Ferromagnet $NdMn_2Ge_2$, ACS Applied Materials and Interfaces **12**, 24125 (2020).

[2] X. Yu, D. Morikawa, T. Yokouchi, K. Shibata, N. Kanazawa, F. Kagawa, T.-h. Arima, and Y. Tokura, Aggregation and collapse dynamics of skyrmions in a non-equilibrium state, Nature Physics **14**, 832 (2018).
